# *Queijo Serra da Estrela* PDO Cheese: Investigation into Its Morpho-Textural Traits, Microbiota, and Volatilome

**DOI:** 10.3390/foods12010169

**Published:** 2022-12-29

**Authors:** Giorgia Rampanti, Ilario Ferrocino, Joanna Harasym, Roberta Foligni, Federica Cardinali, Agnieszka Orkusz, Vesna Milanović, Irene Franciosa, Cristiana Garofalo, Cinzia Mannozzi, Massimo Mozzon, Andrea Osimani, Lucia Aquilanti

**Affiliations:** 1 Department of Agricultural Sciences, Food and Environmental, Marche Polytechnic University, 60131 Ancona, Italy; 2 Department of Agricultural, Forest, and Food Science, University of Turin, Largo Paolo Braccini 2, 10095 Grugliasco, Italy; 3 Department of Biotechnology and Food Analysis, Wroclaw University of Economics and Business, Komandorska 118/120, 53–345 Wrocław, Poland

**Keywords:** lactic acid bacteria, metataxonomic analysis, enzymatic activity, *Lactococcus lactis*, Portuguese cheese

## Abstract

*Queijo Serra da Estrela* is a PDO Portuguese cheese produced through coagulation of raw ewe’s milk using cardoon (*Cynara cardunculus* L.) flowers. The present research was aimed at depicting an up-to-date and comprehensive overview of the microbiota of *Queijo Serra da Estrela* cheese. To this end, viable counting and metataxonomic analysis were carried out on cheeses sampled from four Portuguese artisan producers. Physico-chemical and morpho-textural analyses were also performed, together with the analysis of volatile organic compounds (VOCs). Finally, non-starter lactic acid bacteria (NSLAB) isolated from the cheeses were characterized for their enzymatic activities using a semi-quantitative method. According to the metataxonomic analysis, *Lactococcus lactis* and *Lactococcus piscium* were the species occurring at the highest relative abundance. The isolates collected from the cheeses were assigned to *Enterococcus durans*, *Enterococcus faecalis*, *Enterococcus faecium*, *Enterococcus lactis*, *Levilactobacillus brevis*, *Latilactobacillus graminis*, *Leuconostoc mesenteroides*, and the *Lacticaseibacillus casei* group. The enzymatic characterization of these cultures highlighted esterase, aminopeptidase, acid phosphatase, beta-galactosidase, alpha-glucosidase, and beta-glucosidase among the major enzymatic activities. Fungal populations were dominated by *Debaryomyces hansenii* and *Kurtzmaniella zeylanoides*; however, species rarely found in cheese (e.g., *Candida boidinii, Vishniacozyma victoriae*, and *Starmerella*) were also detected. The volatile compounds characterizing the analyzed cheeses were carboxylic acids and esters, followed by carbonyl compounds and alcohols.

## 1. Introduction

Cheese represents one of the most studied sources of protein that are part of the Mediterranean diet [1]. Indeed, in the Mediterranean biogeographical Region, that includes among others, France, Italy, Greece, Cyprus, Morocco, Tunisia, Spain, and Portugal, a plenty of traditional cheeses is produced [1]. In these countries, traditional cheeses are still manufactured in accordance with ancient traditions that should be preserved and valorized to maintain the uniqueness and quality of these products [2].

Cheese is the result of the coagulation of milk proteins (caseins) through the addition of animal rennet or other milk coagulants (e.g., vegetable rennet) [3]. Cheese is mostly obtained from cow’s, ewe’s, or goat’s milk, whether raw or pasteurized. Among milk coagulants, calf rennet, which is obtained from the fourth stomach of suckling calves, is the most widely exploited at industrial level [4]. In addition, extracts from plants ascribed to *Euphorbia*, *Lactuca*, *Solanum*, *Streblus*, and to members of the Asteraceae family, commonly known as thistles, are used [5]. These latter include various species ascribed to the genera *Carduus*, *Carlina*, *Cirsium*, *Cynara*, *Onopordum*, *Scolymus*, and *Silybum* [5]. Clotting agents play a crucial role in the primary and secondary hydrolysis of milk proteins [5]. After coagulation, the curd is separated from the whey, shaped into cheese moulds or hoops, and in some cases subjected to ripening. The latter stage, resulting in breakdown of acids, fats, and protein, is mainly driven by the activity of pro-technological microorganisms and milk native enzymes [6]. During ripening, flavor, texture, and body characteristics of cheese are developed [6].

The key pro-technological microorganisms spontaneously occurring in the cheese or intentionally added into the milk as primary or secondary starters are represented by lactic acid bacteria. These microorganisms are principally responsible for production of lactic and acetic acid, as well as other minority organic acids such as formic, pyruvic, citric, uric, propionic, or butyric acid [7]; these compounds strongly affect the safety of cheese and its sensory characteristics [8]. Lactic acid bacteria also affect the development of cheese flavor through proteolysis and, hence, the formation of peptides and branched-chain amino acids [9]. As reviewed by Gobbetti et al. [10], primary starters are limited to a small number of lactic acid bacteria species commonly referred to as LAB (e.g., *Lactococcus lactis* subsp. *lactis*, *Streptococcus thermophilus*, and *Lactobacillus helveticus*), whereas other species are included in the group of the so-called non-starter lactic acid bacteria (NSLAB) [10]. The NSLAB strongly affect biochemical and sensory traits of mature cheeses [10].

In the European Union (EU), the quality policy of foods aims to protect the names of specific products to promote their unique characteristics. Among the countries included in the Mediterranean biogeographical Region, Portugal accounts 11 cheeses that have been granted the Protected Designation of Origin (PDO) geographical indication, including: *Queijo de Azeitão*, *Queijo do Pico*, *Queijo de Cabra Transmontano*/*Queijo de Cabra Transmontano Velho*, *Queijo Rabaçal*, *Queijo Terrincho*, *Queijo da Beira Baixa*, *Queijo de Nisa*, *Queijo S. Jorge*, *Queijo Serpa*, *Queijo de Évora*, and *Queijo Serra da Estrela*. This latter has been granted the PDO geographical indication according to Commission Regulation (EC) No 1107/96 [11] and following amendments [12].

*Queijo Serra da Estrela* PDO cheese is produced through coagulation of raw ewe’s milk from Bordaleira Serra da Estrela and/or Churra Mondegueira ewes. The milk is first heated to a temperature between 28 and 32 °C and then clotted using flowers from *Cynara cardunculus* L. Clotting is usually completed in 45–60 min. The curd is then roughly cut and, after the whey has been drained off, it is placed into cheese moulds to obtain the desired shape. Then, a weigh of ~4 to 5 kg is placed on the top of each cheese wheel for about 6 h. In some cases, this latter step is followed by dry salting. The lateral surface of each cheese wheel is then wrapped with a band of white cloth, and the product is left to ripe. The ripening process, which includes two phases, is carried out in chambers where the environmental conditions are maintained as follows: (i) during the first 15–20 days, the cheese is kept at a temperature comprised between 6 and 12 °C and a relative humidity (RU) ranging from 85 to 90%; (ii) between the 20 and 45 h day of ripening, the temperature is maintained between 6 and 14 °C at RU between 90 and 95%. Of note, the minimum ripening time for the popular buttery-type *Queijo Serra da Estrela* PDO cheese is 30 days. The end product is a short regular cylinder with a diameter of 9 to 20 cm, height comprised between 4 and 6 cm, and weight ranging from 0.5 to 1.7 kg, with bulging sides and some bulging on the top. The cheese rind is smooth and semi-soft. The texture is slightly buttery, creamy, and smooth, with few or no eyes. The color is white or yellowish. The taste is smooth, with a slightly acidic bouquet.

To the best of the author’s knowledge, various studies on the microbiology of *Queijo Serra da Estrela* PDO cheese have been performed over the years [13,14,15,16,17,18,19,20]. These studies were mainly carried out using conventional culture-dependent methods and only one recent investigation exploited the potential of metataxonomic analysis to disclose the microbiota of this renowned PDO cheese [21].

Hence, the present research was aimed at depicting an up-to-date comprehensive overview of the microbiota of *Queijo Serra da Estrela* PDO cheeses sampled from four producers located in Portugal. To this end, bacterial and fungal communities naturally occurring in the sampled cheeses were studied through viable counting and metataxonomic analysis. Moreover, a pool of autochthonous NSLAB was isolated from the cheeses, identified, and further characterized for their enzymatic activity using a semi-quantitative method. Finally, physico-chemical and morpho-textural analyses were also carried out on the cheeses, together with the analysis of volatile organic compounds (VOCs).

## 2. Materials and Methods

### 2.1. Sampling of Cheese

Eight samples of *Queijo Serra da Estrela* PDO cheese were collected from four Portuguese artisan producers. Two samples of the same batch were collected from each producer, as follows: samples E1 and E2 from producer 1, located in Vila Nova de Tazem; samples E3 and E4 from producer 2, located in Minhocal; samples E5 and E6 from producer 3 and samples E7 and E8 from producer 4, both producers being in São Cosmado. All sampled cheeses had the same height (~5 cm), diameter (~10 cm), and weight (~500 g). The ripening time was as follows: approximately ~60 days for cheeses collected from producer 1, and 35–40 days for cheeses collected from producer 2, 3, and 4.

No starter cultures were used for the manufacturing of these cheeses. All samples were transported to the laboratory under refrigerated conditions and stored at +4 °C until analysis.

### 2.2. Evaluation of Physico-Chemical Features

A pHmeter (Hanna Instruments, Padova, Italy) equipped with a HI2031 solid electrode (Hanna Instruments) was used for pH determination.

For measurement of total titratable acidity (TTA), 10 g of each sample was added with 90 mL of deionized water and homogenized using a Stomacher 400 Circulator (VWR International PBI, Milan, Italy) at 260 rpm for 5 min. The results were expressed as amount (mL) of 0.1 N NaOH solution needed to titrate the pH of the cheese homogenates back to 8.3 [5].

The cheese content of lactic and acetic acid was determined using the D-/L-Lactic Acid (D-/L-Lactate) (Rapid) test kit and the Acetic Acid (Acetate Kinase Manual Format) test kit (Megazyme, Bray, Ireland), respectively [22].

Water activity (a_w_) was measured using a AquaLab^®^ 3TE analyzer (Decagon Devices, Inc., Pullman, WA, USA) as previously described by Cardinali et al. [5].

Specific volume, expressed as g mL^−1^, was calculated as previously detailed by Cardinali et al. [5].

For each physico-chemical feature considered, the results of three independent measurements were reported as mean ± standard deviation.

### 2.3. Analysis of Morpho-Textural Parameters

Cheese color was assessed using a Konica Minolta CR-310 chroma meter (Ramsey, NJ, USA). Color parameters (L*, a*, b*, chroma, and hue) were taken in triplicate, and expressed as mean ± standard deviation [23].

Cheese texture was evaluated through TPA test (Texture Profile Analysis) using an AXIS texture analyzer FC200STAV500 (AXIS, Gdansk, Poland) provided with the software “AXIS FM” as previously reported by Cardinali et al. [5]. In more detail, an aluminium 20 mm diameter cylindrical probe was used in a double compression test (TPA) to penetrate to 50% depth, at 1 mm s^−1^ speed test. Hardness (*N*) was the force at the maximum deformation, whereas cohesiveness, springiness, chewiness, and resilience were calculated from the peaks. Analysis was carried out in quadruplicate at 25 °C on cheese cylinders (20 mm height and 20 mm diameter) cut from a cheese slice of each sample. The results were reported as mean ± standard deviation.

### 2.4. Microbiological Analyses

Ten grams of each sample were homogenized with 90 mL of sterile peptone water (Oxoid, Basingstoke, UK) in a stomacher apparatus for 2 min at 260 rpm [24]. Serial ten-fold dilutions were prepared from the cheese homogenates and used for viable counting of the following microbial groups: presumptive mesophilic lactococci, presumptive thermophilic streptococci, presumptive mesophilic lactobacilli, coagulase-negative cocci, enterococci, and eumycetes (yeasts and molds). Growth media and incubation temperatures were those already detailed by Cardinali et al. [25].

The results of viable counting, expressed as Log of colony-forming units (cfu) per gram of sample, were reported as mean of two biological replicates ± standard deviation.

### 2.5. DNA Extraction and Sequencing

The E.Z.N.A. soil DNA kit (Omega Bio-tek, Norcross, GA, USA) was used for the extraction of total microbial DNA from the cell pellets obtained by centrifugation of 1 mL of each cheese homogenate (dilution 10^−1^) prepared as previously described. DNA extracts were then checked for quantity and purity using a Nanodrop ND 1000 (Thermo Fisher Scientific, Wilmington, DE, USA), and further standardized using the Qubit ds Kits.

For each sample, the DNA extracts obtained from each biological replicate were pooled to reduce the inter-sample variability [26].

A metataxonomic approach was applied to analyze pooled DNAs. The 16S rRNA gene (V3–V4 regions) was amplified using the primers and procedures described previously [27]. The Illumina guidelines were used for purification of amplicons, taggering and pooling. The Illumina MiSeq platform with V2 chemistry was used to generate 250-bp paired-end reads and the raw *fastq* files obtained were elaborated by QIIME 2 software [28]. *Cutapter* was used to remove prime sequences, and DADA2 algorithm was used to denoise the obtained reads by using the q2-dada2 plugin in QIIME 2 [29]. Taxonomy classification was performed against the *SILVA* database by means of the QIIME2 feature-classifier. The amplicon sequence variants (ASVs) with less than five read counts in at least two samples were excluded to increase the confidence of sequence reads.

The raw read data were deposited in the Sequence Read Archive of NCBI under the bioproject accession number PRJNA822519.

### 2.6. Isolation and Characterization of the Dominant Non-Starter Lactic Acid Bacteria (NSLAB)

#### 2.6.1. Collection of Pure Cultures

Colonies grown on the agar plates used for viable counting of lactic acid bacteria were randomly selected, sub-cultured to purity on the same media used for enumeration and stored at −80 °C for long-term maintenance.

Total genomic DNA was extracted from the collected isolates according to Osimani et al. [30] and checked for purity and quantity using a NanoDrop ND 1000 (Thermo Fisher Scientific, Wilmington, DE, USA). DNA extracts were standardized to a final concentration of 100 ng/μL and subjected to PCR in a Mastercycler X50a (Eppendorf, Hamburg, Germany) using the universal prokaryotic primers 27f and 1495r, as described by Osimani et al. [30]. The amplification was verified by electrophoresis in 1.5% (*w*/*v*) agarose gel and 0.5X Tris/Borate/EDTA (TBE) buffer containing 0.5 µg/mL GelRed^®^ Nucleic Acid Gel Stain, 10,000X in water (Biotium, San Francisco, CA, USA). The amplicons were then sent to Azenta Life Sciences (Leipzig, Germany) for purification and sequencing.

The raw sequences, represented in FASTA format, were analyzed with UCHIME2 software tool to uncover chimeras [31] and were trimmed to remove NNNs and misleading data from the terminations. Afterwards, a BLAST search was exploited to compare the obtained sequences with 16S rRNA sequences of type or reference strains deposited at the GenBank DNA database (http://www.ncbi.nlm.nih.gov/, 12 November 2022). Hence, the sequences of the 60 pure cultures were submitted to the GenBank DNA database to acquire the respective accession numbers.

#### 2.6.2. Semi-Quantitative Assessment of the Enzymatic Activity of NSLAB

The semi-quantitative micromethod API^®^ ZYM (bioMérieux, Marcy-l’Etoile, France) was used for the assessment of the enzymatic activities of the pool of isolates in accordance with the manufacturer’s instructions. Each API^®^ ZYM strip consisted of 20 cupules containing synthetic substrates that were inoculated with each isolate to be assayed. The 20 cupules were designed for the study of the following enzymatic reactions: 1, control; 2, alkaline phosphatase; 3, esterase (C 4); 4, esterase lipase (C 8); 5, lipase (C 14); 6, leucine arylamidase; 7, valine arylamidase; 8, cystine arylamidase; 9, trypsin; 10, alpha-chymotrypsin; 11, acid phosphatase; 12, naphthol-AS-BI-phosphohydrolase; 13, alpha-galactosidase; 14, beta-galactosidase; 15, beta-glucuronidase; 16, alpha-glucosidase; 17, beta-glucosidase; 18, N-acetyl-ß-glucosaminidase; 19, alpha-mannosidase; 20, alpha-fucosidase. The metabolic end products produced during the incubation period were detected through colored reactions revealed by the addition of specific reagents (bioMérieux), as detailed below.

Briefly, a suspension of each isolate in 2 mL of API Suspension Medium was prepared to reach a turbidity of 5–6 McFarland. Sixty-five µL of the resulting suspension was used for the inoculation of each cupule of the API^®^ ZYM strips (bioMérieux). After incubation at 37 °C for 4 h, 1 drop of ZYM A reagent (bioMérieux) and 1 drop of ZYM B reagent (bioMérieux) were added to each cupule. After resting (at least 5 min), the resulting colours were recorded based on intensity and assigned a semiquantitative notation using a colour code ranging from 0 (no color development) to 5 (maximum color intensity); 1 to 4 corresponded to intermediate color intensities, with “3”, “4” and “5” being considered as positive reactions.

### 2.7. Cheese Volatile Profile

Solid phase microextraction (SPME) was used to collect VOCs, as previously described by Belleggia et al. [32]. A Trace 1300 gas chromatograph coupled with a ISQ 7000 single quadrupole mass spectrometer (Thermo Fisher Scientific, Waltham, MA, USA) equipped with a Zebron ZB-5ms capillary column 30 m × 0.25 mm i.d., 0.25 μm film thickness (Phenomenex, Torrance, CA, USA) were used according to the procedure previously detailed by Foligni et al. [33]. According to Mozzon et al. [34], the identification of VOCs was made by matching the mass spectral data with NIST/EPA/NIH Mass Spectral Library 2020, and the chromatographic behaviour with published Kovats retention indices (RIs). An automated spreadsheet was used to simplify the calculation of RIs of the unknown components [35].

### 2.8. Statistical Analysis

Significant differences among cheeses were determined by one-way analysis of variance (ANOVA) using the software JMP^®^ Version 11.0.0 (SAS Institute Inc., Cary, NC, USA) and the Tukey–Kramer’s Honest Significant Difference (HSD) test (*α* = 0.05).

The diversity script of QIIME2 was used to calculate alpha diversity indices. The non-parametric Kruskal–Wallis test in R environment was used to analyze differences between alpha diversity parameters and ASVs frequency.

## 3. Results

### 3.1. Physico-Chemical Characterization

The results of physico-chemical analyses are reported in Table 1.

As for pH, the overall means were comprised between 5.46 and 5.83, with samples of producers 3 and 4 showing the lowest values and those of producers 1 and 2 the highest ones.

Regarding TTA, overall mean values ranged between 7.52 and 13.97 mL of 0.1 NaOH, with samples from producer 1 showing the lowest values and those from producer 3 the highest ones.

As for organic acids, mean content of acetic acid was comprised between 0.11 and 0.27 g/100 g with no statistically significant differences among samples; regarding lactic acid, the overall mean content ranged between 0.63 and 1.13 g/100 g with samples from producer 1 showing the lowest values.

A_w_ was comprised between 0.940 and 0.975, again with samples from producer 1 showing the lowest values.

Finally, mean values for specific volume ranged between 0.98 and 1.11 g/mL, with samples from producer 4 showing the lowest values and those from producer 1 the highest ones.

### 3.2. Morpho-Textural Characterization

The results of color analysis are reported in Table 2.

The average lightness (L*) values varied from 106.6 to 117.8 resulting in significant differences among the producers. The darkest samples were those collected from producer 1, whereas the lightest ones were those collected from producer 2. Differences in tones were also visible in all the samples; cheeses from producers 2 and 3 revealed greenish shades (a*) varying from −0.76 to −2.15. Cheeses sampled from producers 3 and 1 were characterized by red tones up to 1.10. No significant differences were seen in the average values of yellowish shade (b*) of cheeses collected from producers 1, 3, and 4, whereas those from producer 2 were characterized by significantly lower average b* and chroma values.

For hue, cheeses from producers 1 and 4 significantly differed from those sampled from producers 2 and 3.

The results of texture analysis are reported in Table 3.

An overall high level of similarity was seen among cheeses, except for those collected from producer 1, which showed the highest mean value for hardness. Cheeses sampled from producers 1 and 2 differed for cohesiveness, whereas just those from producer 1 showed the highest springiness and chewiness, with mean values of 0.48 and 6.79, respectively. Resilience ranged between 0.16 and 0.18 mean values, with no statistically significant differences among cheeses.

### 3.3. Viable Counting

The results of viable counting are reported in Table 4.

Regarding presumptive lactococci, overall mean counts ranged between 7.98 and 8.66 log cfu/g, with samples from producers 3 and 2 showing the lowest and highest counts, respectively.

Overall mean counts of presumptive thermophilic cocci ranged between 7.16 and 8.28 log cfu/g, with cheeses from producer 1 showing the lowest loads.

As for presumptive lactobacilli, overall mean counts were comprised between 8.13 and 8.50 log cfu/g, with no statistically significant differences among producers.

Coagulase-negative cocci showed overall mean counts comprised between 5.34 and 6.42 log cfu/g, with cheeses from producer 4 showing the highest loads.

Overall mean counts of enterococci ranged between 6.56 and 7.84 log cfu/g, with cheeses from producer 3 and 4 showing the lowest and highest counts, respectively.

As for yeasts, overall mean counts were comprised between 1.27 and 4.24 log cfu/g, with cheeses from producer 3 showing the lowest counts; finally, overall mean counts of molds were always < 1 log cfu/g, except for cheeses sampled from producer 2, whose mold counts attested at 4.07 log cfu/g.

### 3.4. Microbiota Composition

**Figure 1:**
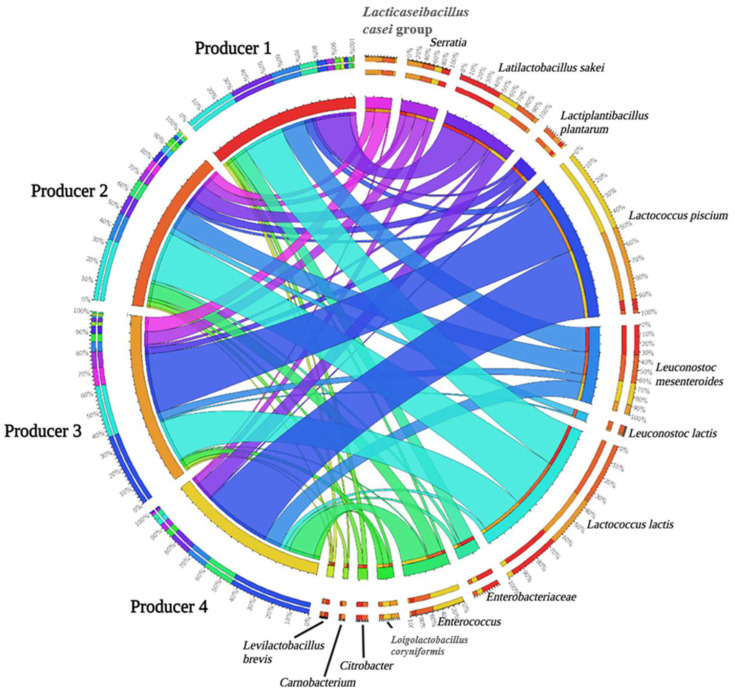
Circular ideogram showing the bacterial distribution (>1% of the relative frequency in at least 2 samples) among the *Queijo Serra da Estrela* PDO cheese PDO cheese samples herein analyzed. Amplicon Sequence Variants (ASVs) and samples are connected with ribbons, whose thickness is proportional to the abundance of ASVs in the connected samples. The outer circle displays the proportion of each ASV in each producer and vice versa.

The incidence (%) of the bacterial taxonomic groups detected in the cheeses herein analyzed is reported in Figure 1 and Supplementary Table S1.

Briefly, *Lactococcus lactis* dominated in almost all cheeses (attesting at ~25–30% of the relative frequency) except for those collected from producer 4, where this ASV was found with the lowest relative frequency (~3%). Other minor ASVs were also detected; these latter included *Leuconostoc mesenteroides* (attesting at 5 to 14% of the relative frequency), *Latilactobacillus sakei* (attesting at 2 to 21% of the relative frequency), *Leuconostoc lactis* (attesting at 1 to 4% of the relative frequency), and *Serratia* (attesting at 6 to 8% of the relative frequency) were found in cheeses from producers 2 and 3, whereas *Lactococcus piscium* was detected in cheeses from producers 3 and 4 (attesting at 37 to 42% of the relative frequency). *Enterococcus* was also detected in cheeses from producer 4, at a high relative frequency (~16%).

**Figure 2:**
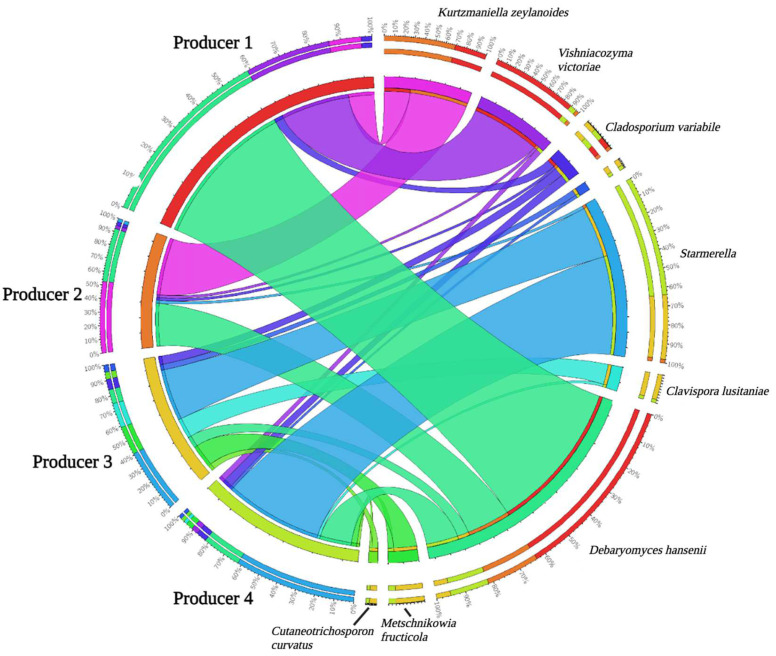
Circular ideogram showing the fungal distribution (>1% of the relative frequency in at least 2 samples) among the *Queijo Serra da Estrela* PDO cheese PDO cheese samples herein analyzed. Amplicon Sequence Variants (ASVs) and samples are connected with ribbons, whose thickness is proportional to the abundance of ASVs in the connected samples. The outer circle displays the proportion of each ASV in each producer and vice versa.

The incidence (%) of the fungal taxonomic groups is reported in Figure 2 and Supplementary Table S2.

In more detail, samples from producer 1 were characterized by the presence of *Debaryomyces hansenii*, *Kurtzmaniella zeylanoides*, and *Vishniacozyma victoriae* (attesting at 52, 9, and 24% of the relative frequency, respectively). Samples from producer 2 were characterized by the presence of *Galactomyces geotrichum*, *K. zeylanoides*, *Candida boidinii*, *D. hansenii*, and *Naganishia albidosimilis* (attesting at 30, 20, 15, 15, and 10% of the relative frequency, respectively). Samples from producer 3 were characterized by the presence of *Starmerella*, *Metschnikowia fructicola*, *Clavispora lusitaniae*, *Protomyces inouyei*, and *D. hansenii* (attesting at 18, 8, 7, 5, and 4% of the relative frequency, respectively). Finally, cheeses sampled from producer 4 were characterized by the presence of *Starmerella*, *D. hansenii*, *Pichia fermentans*, *Cladosporium variabile*, and *M. fructicola* (34, 11, 4, 3, and 2% of the relative frequency, respectively).

### 3.5. Isolation Campaign and Semi-Quantitative Assessment of Enzymatic Activity of Pure Cultures

The closest relatives, the percent identities, and the accession numbers of the sequences obtained from the pool of NSLAB isolated from the cheeses are reported in Table 5.

In detail, enterococci represented the most frequently isolated NSLAB (12 isolates), with *Enterococcus durans* being the most abundantly detected species (6 isolates), followed by *Enterococcus faecalis* (4 isolates), *Enterococcus faecium* (1 isolate), and *Enterococcus lactis* (1 isolate). *Levilactobacillus brevis* was also isolated (7 isolates), together with *Lacticaseibacillus casei* group (6 isolates), *Latilactobacillus graminis* (3 isolates), and *Leuconostoc mesenteroides* subsp. *dextranicum* (2 isolates).

The results of semi-quantitative assessment of the enzymatic activities of the pool of isolates are reported in Table 6.

According to the manufacturer’s instructions, only the isolates that showed a positive reaction (coded as “3”, “4”, or “5”) were considered as positive for the tested enzymatic activity.

Regarding enterococci, just one *E. faecalis* isolate (SE12) showed the esterase (C 4) activity, whereas two isolates of *E. durans* (SE28 and SE29) were positive for esterase lipase (C 8); the same isolates were also positive for leucine arylamidase and beta-glucosidase. SE7 was positive for acid phosphatase, whereas SE22 was positive for naphthol-AS-BI-phosphohydrolase. Six isolates being SE5, SE6, SE13, SE14, SE16, and SE28 showed a positive reaction for beta-galactosidase, whereas SE7 and SE12 were positive for alpha-glucosidase.

As for *L. brevis*, six out of seven isolates, being SE2, SE3, SE11, SE19, SE20, and SE24, were positive for leucine arylamidase, whereas the same isolates but SE20 were also positive for valine arylamidase and acid phosphatase. SE3, SE11, SE19, and SE24 were positive for naphthol-AS-BI-phosphohydrolase, whereas the same isolates but SE19 were also positive for alpha-galactosidase. All the *L. brevis* isolates were positive for beta-galactosidase and beta-glucosidase, whereas the isolates SE19 and SE24 were positive for beta-glucuronidase; finally, SE11, SE20, and SE24 were also positive for alpha-glucosidase.

Regarding the *L. casei* group, three out of six isolates, being SE8, SE9, and SE17, were positive for esterase (C4), whereas the same isolates but SE9 were also positive for esterase lipase (C8). Five out of six isolates belonging to this clade, namely SE8, SE10, SE17, SE25, and SE30, showed a strong leucine arylamidase activity, whereas all the six isolates assayed were positive for valine arylamidase. SE25 was also positive for cystine arylamidase; SE10, SE17, and SE25 for acid phosphatase; and SE8, SE9, SE10, SE17, and SE25 for naphthol-AS-BI-phosphohydrolase. Finally, the isolates SE8, SE25, and SE30 showed a positive reaction for beta-galactosidase, whereas the isolates SE17 and SE25 were positive for alpha-glucosidase, and only SE17 was positive for beta-glucosidase.

As for *L. graminis*, only the isolate SE23 was positive for alkaline phosphatase, whereas all the three isolates SE4, SE23, and SE26 were positive for leucine arylamidase and valine arylamidase.

Finally, only one out of the two *L. mesenteroides* subsp. *dextranicum* isolates (SE21) was positive for esterase (C 4), whereas SE27 was positive for alpha-galactosidase. Both these isolates were positive for beta-galactosidase, whereas only SE27 was positive for beta-glucosidase.

No isolates showed a positive reaction for lipase (C14), trypsin, N-acetyl-ß-glucosaminidase, alpha-mannosidase, or alpha-fucosidase, whereas only few isolates showed a very weak activity (<3) for alpha-chymotrypsin.

### 3.6. Volatile Profile

The detailed identification of the VOCS found in the cheeses is reported in Table 7.

## 4. Discussion

### 4.1. Physico-Chemical and Morpho-Textural Traits of Cheeses

Regarding pH, data were comparable with those already reported by Fogeiro et al. [14] and Macedo et. al. [15] for the same cheese, with values comprised between 5.24 and 5.30. The reduction of pH during cheese manufacturing is principally due to the metabolic activity of lactic acid bacteria producing organic acids through fermentation of lactose. Of note, acidification improves sensory profile and safety of cheeses, with a remarkable reduction in the load of pathogenic (e.g., *Enterobacteriaceae* and *Staphylococcaceae*) or spoilage (e.g., *Pseudomonadaceae*) microorganisms naturally occurring in the raw milk [36]. pH is strictly related with TTA; this latter parameter represents a better predictor than pH of how organic acids impact on flavor of foods. Unlike strong acids that are fully dissociated, organic acids are only partially ionized. While pH measures the concentration of free protons in a solution, total titratable acidity measures the sum of free protons and undissociated acids [37]. In cheeses, TTA is influenced by organic acids produced during fermentation as well as by milk proteins and acid phosphates [38]. In the present study, a high variation for TTA was seen among producers, although, to the author’s knowledge, no previous studies reporting TTA values of *Queijo Serra da Estrela* PDO cheese are currently available for further comparison of data. In any case, the TTA values herein measured were slightly lower than those reported by Cardinali et al. [5,25,39] for the Portuguese thistle-curdled cheeses *Queijo de Azeitão* PDO, *Queijo de Nisa* PDO and *Queijo da Beira Baixa* PDO.

In the cheeses herein analyzed, as expected, lactic and acetic acids prevailed among organic acids. The release of lactic acid is principally due to the metabolism of homofermentative lactic acid bacteria species, whereas acetic acid is mostly produced by heterofermentative lactic acid bacteria species [40]. Together with the already mentioned protective effect against pathogenic and spoilage bacteria [41], lactic and acetic acid contribute to cheese aroma formation. In the *Queijo Serra da Estrela* PDO cheeses herein analyzed levels of lactic acid were notably higher than those of acetic acid, thus confirming the prevalence of homofermentative species. To the authors’ knowledge, no data are available on the content of these two organic acids in *Queijo Serra da Estrela* PDO cheese for further comparison of the results. However, the levels herein recorded for these two organic acids were higher than those found in *Queijo de Azeitão* PDO [39], and comparable to those found in *Queijo de Nisa* PDO and *Queijo da Beira Baixa* PDO [5,25].

Regarding a_w_, this parameter is strongly affected by ripening; together with pH, it represents a key parameter for cheese preservation. The differences emerged among cheeses might reflect the slight variations in ripening time and conditions at the four dairy plants. However, again no previous data on a_w_ of *Queijo Serra da Estrela* PDO cheese are available for further comparison of results.

The evaluation of specific volume of cheese allows the presence of any porous or microporous structure to be revealed. Although *Queijo Serra da Estrela* PDO is recognized as a cheese with no or low porosity, some differences were seen in the specific volume of cheeses herein analyzed, with samples from producer 1 being characterized by the highest specific volume, feasibly due to the longer ripening time (approximately 60 days) in respect with the other sampled cheeses.

For cheese color and texture, both parameters depend on multiple factors, including the origin of milk, the pasture location, and the grazing seasons [25]. All *Queijo Serra da Estrela* PDO cheeses herein analyzed were characterized by comparable color and texture, except for those sampled from producer 1, which where darker and harder than those sampled from the other three producers. Again, this finding might be ascribed to the longer maturation of cheeses from producer 1.

### 4.2. Bacterial Biota

Microbial counts highlighted the occurrence of viable populations of thermophilic and mesophilic cocci as well as mesophilic lactobacilli. The contribution of these microbial groups to cheese fermentation and ripening is well acknowledged. First, as already mentioned, these pro-technological bacteria are responsible for fermentation of lactose into lactic acid and other secondary metabolites. Moreover, lactic acid bacteria can improve the rheological, textural, microstructural, and sensory properties of cheese by producing long-chain polysaccharides composed of repeated units of mono-sugars or their derivatives [42]. These microorganisms can also contribute to enhancing the safety of cheese by producing bacteriocins; these latter are peptides with bacteriostatic, bactericidal, and/or bacteriolytic effect [43]. Finally, the enzymatic activities exerted by lactic acid bacteria strongly affect the taste and flavour of cheese [9].

The counts of these microorganisms in the *Queijo Serra da Estrela* PDO cheeses herein analyzed were in accordance with those reported by Tavaria and Malcata [16] for the same cheese.

Although enterococci are considered as members of this heterogeneous bacterial group, their role in cheese is still controversial since strains within the genus *Enteroccocus* can carry transferable antibiotic resistances or virulence genes [44]. Moreover, some strains can cause bacteremia, endocarditis, and other infections in humans [44]. However, in past studies, enterococci isolated from cheese whey showed a high biotechnological potential as starter cultures [44]. As even reviewed by Foulquié Moreno et al. [45], in Mediterranean cheeses produced with raw ewes’ or goats’ milk, enterococci contribute to the typical taste and flavor of cheeses through proteolysis, lipolysis, and citrate breakdown, thus explaining the interest of the dairy industry for this bacterial group [46]. The viable counts of enterococci in *Queijo Serra da Estrela* PDO cheeses herein analyzed were in accordance with those reported by Tavaria and Malcata [16] in the same cheese, thus confirming the importance of this bacterial group in such a Mediterranean cheese.

Regarding the coagulase-negative cocci, as reviewed by Khusro and Aarti [47], these microorganisms can improve the color and flavor of cheese during ripening, through lipolysis and proteolysis [47]. To the author’s knowledge, the scientific literature on staphylococci in *Queijo Serra da Estrela* PDO cheese only deals with the occurrence of coagulase-positive species; hence, data obtained in the present study represent an advancement in the field. However, a massive presence of coagulase-negative cocci has already been detected in other Portuguese cheeses clotted with vegetable rennet [5,25,39], thus suggesting a role of this bacterial group in these cheeses.

Metataxonomic analysis undoubtedly represents a powerful tool to highlight the relative abundance of bacterial and fungal taxa in cheeses. Surprisingly, only one study has applied a metataxonomic approach to the investigation of *Queijo Serra da Estrela* PDO cheese microbiota so far [21], hence, the results of the present investigation undoubtedly represent an advancement in the knowledge of the microbial species harbored by this renowned Portuguese cheese.

Among the most represented ASVs, *L. lactis* was detected in all the analyzed samples. *L. lactis* is a mesophilic spherical or ovoid-shaped bacterium that can grow at 10 °C but not at 45 °C; it is considered a key species in fermented dairy products since it produces lactic acid from lactose and contributes to the breakdown of milk proteins during fermentation [48,49]. The metabolic activity of *L. lactis* is also essential for flavor formation through the conversion of amino acids into volatile aroma compounds [50]. Finally, *L. lactis* contributes to refine the texture characteristics of cheese being able to produce exopolysaccharides, these latter directly affecting the rheological properties of cheeses [50]. The occurrence of this species in both Portuguese and Italian thistle-curdled cheeses is well documented [5,25,39,51,52]. Regarding *Queijo Serra da Estrela* PDO cheese, strains ascribed to *L. lactis* had previously been isolated [53,54]; this species was also detected by Rocha et al. [21] and Tavaria & Malcata [19] by sequencing of rRNA gene amplicons, thus confirming the crucial role of this microorganism in fermentation and ripening of *Queijo Serra da Estrela* PDO cheese.

As for leuconostocs, the presence of these bacteria in cheese is mainly related to contamination from the dairy environment, being considered as NSLAB [55]. In cheese, leuconostocs are responsible for the development of flavor compounds as diacetyl and acetoin. A synergistic relationship between *Leuconostoc* and *L. lactis* is usually established in cheese, since *Leuconostoc* metabolizes citrate and produces flavor compounds under acidic pH, this latter affected by the lactic acid production by *L. lactis* [55]. Some *Leuconostoc* strains are also able to produce heat-stable bacteriocins (e.g., mesentericin) active against *Listeria* spp. [55]. Moreover, *L. mesenteroides* is known to produce exopolysaccharides (e.g., dextran) with texturizing function (e.g., increase of viscosity and strengthening of casein network) [55].

*L. sakei* is one of the key species in meat fermentation [56]; hence, its high occurrence in the cheeses herein analyzed is quite uncommon and requires further investigation. However, closest relatives to *L. sakei* have already been detected by Aquilanti et al. [51] in a *Caciotta* cheese manufacture curdled with *C. cardunculus* L., as well as in other Portuguese cheeses produced with vegetable rennet, as *Queijo de Nisa* PDO, *Queijo da Beira Baixa* PDO, and *Queijo de Azeitão* PDO [5,25,39], with a possible role of this microorganism as a NSLAB species [57].

As for *L. piscium*, this *Lactococcus* species is usually detected among spoilage microorganisms in packaged meat, although recent insights into the microbiota of Portuguese thistle-curdled cheeses reported its presence among the autochthonous NSLAB [5,25,39]. Interestingly, no ASVs belonging to *L. piscium* had previously been detected in *Queijo Serra da Estrela* PDO by Rocha et al. [21] using a metataxonomic approach, thus confirming the need for further metataxonomic studies on this Portuguese cheese to better disclose its biodiversity.

*Enterococcus* was massively detected in *Queijo Serra da Estrela* PDO cheeses from producer 4 and, to a lesser extent, from the other producers. The occurrence of enterococci in *Queijo Serra da Estrela* PDO cheese had already been reported by Foulquié Moreno et al. [45] and Rocha et al. [21], the latter authors using a metataxonomic approach; members of the genus *Enterococcus* have also been found by several authors in thistle-curdled cheeses [5,25,39,51,52,58]. In the present study, *Enterococcus* spp. represented the most frequently isolated NSLAB whose enzymatic activities will be thoroughly discussed below.

#### 4.2.1. Enzymatic Activity of Lactic Acid Bacteria Isolates

The pool of isolates collected from the cheeses herein analyzed reflected the occurrence of some NSLAB found via metataxonomic analysis (e.g., *Leuconostoc mesenteroides*, *Enterococcus*, *Lacticaseibacillus*). For most of these isolates, an unambiguous classification to the species level was achieved, whereas 6 isolates could be just assigned to the *Lacticaseibacillus casei* group. For this latter clade, which includes *Lacticaseibacillus casei*, *Lacticaseibacillus paracasei*, and *Lacticaseibacillus rhamnosus* (basonym *Lactobacillus casei*, *Lactobacillus paracasei*, and *Lactobacillus rhamnosus*, respectively) the analysis of the 16S rRNA gene does not allow unknown isolates to be identified to the species level [59,60].

The semi-quantitative assessment of the enzymatic activities of the isolates allowed a better comprehension of the bacterial interaction with the cheese matrix to be obtained.

##### *Enterococcus* spp.

As for *E. faecalis* SE12, which was positive for esterase (C 4), this enzymatic activity has already been observed by Cebrián et al. [61] in one *E. faecalis* strain isolated from Spanish raw ewe’s milk cheese. Esterase is a hydrolytic enzyme that is involved in the enterococci life cycle (e.g., to provide nutrients, to assist in biofilm formation, and to start the proinflammatory responses) [62]. In cheese, esterases produced by *E. faecalis* promote the transformation of fats into fatty acids and glycerol [61].

The isolate *E. faecalis* SE7 was positive for acid phosphatase; this enzyme is a non-specific hydrolase that liberates phosphate ions from organic esters at pH values ranging from ∼4.5 to 6.0 and is essential for the hydrolysis of phosphopeptides during cheese ripening [63,64]. Of note, the activity of this enzyme has already been found in *E. faecalis* strains isolated from *Pecorino Abruzzese* cheese [64].

The alpha-glucosidase activity was observed in only two *E. faecalis* isolates herein assayed. This hydrolase was among the most active enzymes in *E. faecalis* strains isolated from ewe’s milk *Roncal* and *Idiazabal* cheeses (Spain) [65].

As for the isolates *E. durans* SE28 and SE29, the esterase lipase (C 8) activity was observed. In this regard, Tsanasidou et al. [66] have already reported the strong esterase-lipase activity of *E. durans* strains isolated from Greek cheese. The presence of esterase lipase is a beneficial trait during cheese ripening for production of cheese flavors. Both the above-mentioned isolates were also positive for leucine arylamidase and beta-glucosidase activity. Leucine arylamidase is an aminopeptidase that has already been detected in enterococci isolated from artisanal productions of Azorean *Pico* cheese [67], whereas beta-glucosidase is an enzyme belonging to the hydrolases class, whose presence has already been reported in enterococci [68]. This enzyme acts on the beta-glycosidic bonds of polysaccharides, hydrolyzing the terminal residues of beta-D-glucose.

Finally, most of the isolated enterococci showed a strong beta-galactosidase activity. This glycosidase, usually known as lactase, catalyzes the hydrolysis of the beta-glycosidic bond between glucose and galactose [69], thus suggesting the high adaptation of the assayed *Enterococcus* isolates to the dairy environment.

##### *Levilactobacillus brevis* 

*L. brevis* represents one of the most common NSLAB species detected in Mediterranean and Middle Eastern cheeses obtained from raw ewes’ and goats’ milk [70].

In the present study, most isolates ascribed to *L. brevis* were positive for leucine arylamidase and valine arylamidase. The presence of these aminopeptidases has already been reported for this species by Son et al. [71] and Song et al. [72].

The acid phosphatase showed by most of the *L. brevis* isolates herein assayed has already been reported for the same species by Okoth et al. [73].

A great part of these *L. brevis* isolates was also positive for alpha-galactosidase. This enzyme is an exoglycosidase that hydrolyzes the terminal alpha-galactosyl portions from glycoproteins, glycolipids, galactomannans, and galactolipids [74]. Moreover, alpha-galactosidase allows bacteria to use polysaccharides as a carbon source since it cleaves the alpha-1,6 bond between galactose and glucose [74,75].

As for lactase (beta-galactosidase), all the tested *L. brevis* isolates showed this enzymatic activity, although this heterofermentative species rarely ferments milk.

Regarding beta-glucosidase, this enzymatic activity has already been observed in *L. brevis* strains of vegetable origin [71,72]. Beta-glucosidase and beta-glucuronidase can act as carcinogens [76], thus suggesting the need for further investigations into *L. brevis* isolates that exhibited these enzymatic activities.

As for the alpha-glucosidase activity found in the isolates *L. brevis* SE11, SE20, and SE24, it is known that, in this species, this enzyme is mainly found in the intracellular form, and it principally degrades disaccharides and oligosaccharides [77].

##### *Lacticaseibacillus casei* Group

As for esterase activity showed by some isolates ascribed to the *L. casei* group, it is noteworthy that Fenster et al. [78] have already characterized intracellular esterase from *L. casei*. Hence, the same authors suggested that this enzyme might play a crucial role in cheese flavor development by modulating ester profiles in cheese.

The isolates ascribed to the *L. casei* group were also positive for the leucine arylamilase and valine arylamilase activity; both these enzymatic activities have already been observed by Colombo et al. [79] in *L. casei* isolates collected from the dairy environment. In the study herein carried out, the low number of isolates positive for cystine arylamilase was in accordance with previous investigations reporting a weak or even no activity for dairy isolates ascribed to this clade [79,80]. Some isolates ascribed to the *L. casei* group herein assayed were also positive for acid phosphatase; of note, the production of this latter enzyme in *L. casei* has already been reported by Colombo et al. [79]. Acid phosphatase of *L. casei* can be useful in metabolizing phosphates in the acidic environment occurring in cheese during maturation [81].

Finally, half of the *L. casei* group herein assayed showed beta-galactosidase. As observed by de Souza et al. [82], beta-galactosidase activity in *L. casei* is strain depended; this finding likely explains the absence of this activity in all the *L. casei* group isolates obtained from *Queijo Serra da Estrela* PDO cheeses.

##### *Latilactobacillus graminis* 

*L. graminis* was the sole isolate that showed a positive reaction for alkaline phosphatase. This hydrolase, whose activity has widely been reported in lactic acid bacteria, removes phosphate groups from various molecules, such as nucleotides, proteins, and alkaloids [83].

All isolates of *L. graminis* also showed leucine arylamidase and valine arylamidase activity. To the author’s knowledge, a paucity of data regarding these enzymatic activities in *L. graminis* is available in the scientific literature for further comparison of data. However, the presence of these aminopeptidases suggests a contribution of this NSLAB species to cheese flavor formation.

##### *Leuconostoc mesenteroides* 

As for *L. mesenteroides*, only one isolate showed esterase activity; this result is in accordance with that reported by González et al. [84], who found weak or no esterolytic activity in leuconostocs used as starters to produce *Genestoso* cheese, a traditional dairy product from the North of Spain.

The alpha-galactosidase activity showed by the isolate *L. mesenteroides* SE27 was in accordance with the results obtained by Kamarinou [80] that observed the same enzymatic activity in autochthonous *L. mesenteroides* strains isolated from Greek traditional dairy products.

As for beta-galactosidase, the presence of this enzymatic activity in *Leuconostoc* depends on the strain or the substrate (glucose or lactose) [85]. Huang et al. [85] reported that the optimum pH for *L. mesenteroides* beta-galactosidase was comprised between 7.2 and 7.5; the same authors also observed an inhibitory effect of Ca^2+^ on the activity of this enzyme, depending on the CaC1_2_ concentration.

### 4.3. Mycobiota

In artisanal cheeses, yeasts constitute an important part of the microbiota since they grow well in acidified environments and do usually not compete with bacteria for nutrients [86]. As reviewed by Bintsis [86], in cheese, yeasts exert esterase or lipase activity as well as peptidase activity, thus contributing to flavor formation. Moreover, yeasts use lactose produced by lactic acid bacteria with a subsequent increase of pH in cheeses [86].

To the authors’ knowledge, to date, yeast populations of *Queijo Serra da Estrela* PDO cheese have only partly been investigated; hence, the present study represents a further advancement in the knowledge of the mycobiota occurring in this dairy product.

Although with wide variations, likely due to the artisanal practice, the counts of yeasts observed in cheeses herein analyzed were in accordance with those reported by Freitas and Malcata [87] in the same cheese, attesting at about 3–4 log cfu/g.

Regarding molds, only cheeses from producer 2 were characterized by high viable counts, whereas the remaining cheeses showed loads < 1 log cfu/g. To the author’s knowledge, no data on molds occurring in *Queijo Serra da Estrela* PDO cheese are available in the scientific literature for further comparison of results.

In the present study, the metataxonomic analysis of fungal populations allowed major and minor taxa to be detected.

Regarding *D. hansenii*, this species advantages of low a_w_, acidic environment, and high salt content, thus explaining its dominance over other yeasts [88]. *D. hansenii* has a high proteolytic activity (casein hydrolysis) and a moderate lipolytic activity, being able to hydrolyze palmitic acid and stearic acid esters and, to a lesser extent, oleic acid ester [88]. The occurrence of *D. hansenii* in PDO cheeses manufactured from ovine and caprine milks in the Iberian Peninsula, including *Queijo Serra da Estrela* PDO, has already been reported [5,21,25,39,87].

*V. victoriae* is a yeast species able to grow at low temperatures; moreover, it attracted the attention of the food industry for its ability to secrete killer toxins and hydrolytic enzymes (protease, chitinase, and glucanase) [89]. Furthermore, *V. victoriae* can grow using lactose, thus explaining its presence in the analyzed cheeses [89]. To the authors’ knowledge, *V. victoriae* has never been detected in *Queijo Serra da Estrela* PDO cheese before, although it has already been identified among the minority yeasts occurring in *Queijo da Beira Baixa* PDO [25].

As for *Naganishia*, this extremophilic fungus, previously ascribed to the *Cryptococcus albidus* clade, was found to dominate the eumycete populations of soil-like material (tephra) on volcanoes [90]. To the authors’ knowledge, *N. albidosimilis* has never been detected in cheese, before, although *Cryptococcus* species have already been identified in milk as well as surface mold-ripened and blue cheeses [86].

*G. geotrichum* is a mold that can be found in soil, plants, and fruits [91]. This microorganism is also part of the autochthonous mycobiota inhabiting dairy products, since it has been detected in raw milk and on the surface of semi-hard, mold-ripened, and semi-soft cheeses [92]. Interestingly, Grygier et al. [92] have recently observed a high polyunsaturated fatty acids (PUFA) production carried out by strains of *G. geotrichum*, thus suggesting the use of this mold to naturally enrich cheeses in PUFA. To the authors’ knowledge, the presence of *G. geotrichum* has never been reported in *Queijo Serra da Estrela* PDO cheese before; however, its occurrence in *Queijo de Azeitão* PDO cheese has been reported by Cardinali et al. [39].

In fermented dairy products, *Starmerella* has rarely been detected, although this microorganism has already been identified among the minority species occurring in *Queijo de Azeitão* PDO and *Queijo da Beira Baixa* PDO cheeses [25,39]. Of note, the extracellular biosurfactants (sophorolipids) produced by *Starmerella* spp. showed to be active against pathogenic foodborne fungi [93], thus suggesting the need for further investigation on the contribution of this yeast for improvement of cheese safety.

*C. boidinii* has rarely been detected in cheese; notwithstanding, the presence of this yeast has already been reported by Papademas and Robinson [94] in mature commercial *Halloumi* cheeses. The enzymatic profile of *C. boidinii* isolates collected from *Halloumi* cheeses showed alkaline phosphatase, esterase (C4), esterase lipase (C8), leucine aminopeptidase, acid phosphatase, and phosphoamidase activity [94], thus suggesting a key role of this yeast in cheese flavor formation. *C. boidinii* has also been detected among the minority yeasts in *Queijo de Azeitão* PDO cheese [39].

Finally, *K. zeylanoides* (formerly known to as *Candida zeylanoides*) has already been detected in *Queijo Serra da Estrela* PDO by Rocha et al. [21]. As suggested by Rocha et al. [21], the occurrence of this yeast in cheese might be ascribed to latent infection (mastitis) of lactating bovine females, thus suggesting the need for a constant monitoring of the health conditions of these dairy animals.

### 4.4. Volatile Organic Compounds

Regarding the short-chain fatty acids, butanoic, hexanoic, 2-methyl and 3-methyl butanoic are usually recognized as key molecules in the characterization of the aroma profile of different cheese varieties, due to their low perception threshold [95,96].

Of note, most linear acids and branched-chain fatty acids (e.g., 2-methylpropanoic, 3-methylbutanoic and 2-methylbutanoic) are produced through lipolysis, microbial fermentation, and amino acid metabolism occurring during cheese maturation [97].

Moreover, aldehydes, esters, and methyl ketones greatly contribute to the production of other aroma compounds [98]. In the *Queijo Serra da Estrela* PDO cheeses herein analyzed, 2-butanone was the most abundant methyl ketone detected, thus suggesting a role of this compound to the enrichment of cheese aroma with a butterscotch odor note. Methyl ketones were among the most abundant compounds identified in *Queijo da Beira Baixa* PDO cheese and *Cheddar* [25,81].

In the present study, phenylacetaldehyde and benzaldehyde might likely be originated by the microbial metabolism of phenylamine and tryptophan [99].

The occurrence of isovaleric acid (which is a product of leucine catabolism) in the samples herein analyzed was in accordance with what reported by Tavaria et al. [100] about the abundance of volatile fatty acids in *Queijo Serra da Estrela* PDO cheese.

Ester fraction was mainly represented by ethyl esters of fatty acids as ethyl esters of octanoic and decanoic acids, which confer floral and fruity notes to the analyzed cheeses.

The high amounts of alcohols detected in the *Queijo Serra da Estrela* PDO cheeses herein analyzed were in accordance with those already found in other Portuguese cheeses [5,25]. It is well known that ethanol, which is responsible for the formation of esters, is produced by heterofermentative lactic acid bacteria and its contribution to the aroma is generally limited.

## 5. Conclusions

Raw milk PDO cheeses represent a source of still partly undisclosed microbial diversity, where well known microbial species driving fermentation co-exist with other co-occurring microorganisms that are likely to have a strong impact in the sensory traits of cheeses. In the present study, *L. lactis* and *L. piscium* represented the lactic acid bacteria detected at the highest relative abundance in *Queijo Serra da Estrela* PDO cheese. At this regard, the leading role of *L. lactis* in cheese fermentation is widely acknowledged, whereas the influence of *L. piscium* in cheese maturation has still to be clarified. Moreover, *E. durans*, *E. faecalis*, *E. faecium*, *E. lactis*, *L. brevis*, *L. casei* group, *L. graminis*, and *Leuconostoc mesenteroides* subsp. *dextranicum* were isolated from the cheeses. The enzymatic characterization of the pool of NSLAB allowed the comprehension of the role that these microorganisms might play in the definition of sensory and volatile traits of *Queijo Serra da Estrela* PDO cheese. The results overall collected suggested the need for further investigations on the pro-technological features (e.g., production of bacteriocins and exopolysaccharides) of the isolates for their possible exploitation as adjunct cultures in cheese manufacturing. In the cheeses herein analyzed, fungal populations were dominated by *D. hansenii* and *K. zeylanoides*, however species rarely found in cheese (e.g., *C. boidinii, V. victoriae*, and *Starmerella*) were surprisingly detected, thus representing an advancement in the knowledge of the mycobiota naturally occurring in *Queijo Serra da Estrela* PDO cheese. The volatile compounds characterizing the analyzed cheese were carboxylic acids and esters, followed by carbonyl compounds and alcohols.

Differences among most of the morpho-textural traits were observed in cheeses collected from producer 1 in respect with those collected from the other producers. The observed differences were likely affected by the prolonged ripening time of cheeses from producer 1.

The data overall collected allowed an up-to-date and a comprehensive overview of morpho-textural traits, microbiota, and volatilome of *Queijo Serra da Estrela* PDO cheese, thus contributing to improve the knowledge of this renowned Portuguese cheese and providing objective quality parameters for product evaluation and valorization.

## Figures and Tables

**Table 1:** Results of physico-chemical analysis of *Serra da Estrela* PDO cheeses.

Cheese Source	pH	Total Titratable Acidity (mL of 0.1N NaOH)	Acetic Acid (g 100 g^−1^)	Lactic Acid (g 100 g^−1^)	Water Activity	Specific Volume (g mL^−1^)
Producer 1	5.83 ± 0.06 ^a^	7.52 ± 1.44 ^c^	0.11 ± 0.04 ^a^	0.63 ± 0.09 ^b^	0.940 ± 0.011 ^b^	1.11 ± 0.14 ^a^
Producer 2	5.71 ± 0.18 ^a^	9.82 ± 2.90 ^b^	0.12 ± 0.03 ^a^	1.01 ± 0.09 ^a^	0.975 ± 0.003 ^a^	1.09 ± 0.05 ^ab^
Producer 3	5.54 ± 0.09 ^b^	13.97 ± 0.41 ^a^	0.27 ± 0.09 ^a^	1.13 ± 0.11 ^a^	0.969 ± 0.004 ^a^	1.07 ± 0.11 ^ab^
Producer 4	5.46 ± 0.09 ^b^	11.80 ± 1.74 ^ab^	0.26 ± 0.05 ^a^	1.08 ± 0.04 ^a^	0.963 ± 0.008 ^a^	0.98 ± 0.06 ^b^

Within each column, overall means with different superscript letters are significantly different (*p* < 0.05).

**Table 2:** Results of color analysis of *Queijo de Estrella* PDO cheeses.

Cheese Source	L*	a*	b*	Chroma	Hue
Producer 1	106.6 ± 3.6 ^c^	0.35 ± 0.77 ^a^	40.54 ± 1.47 ^a^	40.55 ± 1.47 ^a^	89.58 ± 1.07 ^b^
Producer 2	117.8 ± 1.7 ^b^	−0.90 ± 0.21 ^bc^	34.00 ± 1.23 ^b^	34.01 ± 1.23 ^b^	91.43 ± 0.3 ^a^
Producer 3	116.0 ± 1.9 ^ab^	−1.86 ± 0.67 ^c^	39.42 ± 0.97 ^a^	39.46 ± 0.99 ^a^	92.65 ± 0.93 ^a^
Producer 4	113.3 ± 1.8 ^a^	0.20 ± 1.04 ^ab^	39.24 ± 2.16 ^a^	39.25 ± 2.15 ^a^	89.63 ± 1.48 ^b^

L*—lightness from black (0) to white (100), a*—green (−) to red (+), b*—blue (−) to yellow (+). Within each column, overall means with different superscript letters are significantly different (*p* < 0.05).

**Table 3:** Results of texture analysis of *Queijo de Estrella* PDO cheeses.

Cheese Source	Hardness	Cohesiveness	Springiness	Chewiness	Resilience
Producer 1	53.36 ± 15.67 ^a^	0.22 ± 0.04 ^b^	0.48 ± 0.18 ^a^	6.79 ± 5.83 ^a^	0.16 ± 0.03 ^a^
Producer 2	7.99 ± 1.05 ^b^	0.30 ± 0.07 ^a^	0.28 ± 0.07 ^b^	0.65 ± 0.12 ^b^	0.18 ± 0.01 ^a^
Producer 3	15.30 ± 1.52 ^b^	0.25 ± 0.03 ^ab^	0.26 ± 0.05 ^b^	0.95 ± 0.19 ^b^	0.16 ± 0.03 ^a^
Producer 4	11.89 ± 0.94 ^b^	0.26 ± 0.04 ^ab^	0.28 ± 0.04 ^b^	0.83 ± 0.09 ^b^	0.17 ± 0.02 ^a^

Within each column. overall means with different superscript letters are significantly different (*p* < 0.05).

**Table 4:** Viable counts of *Serra da Estrela* PDO cheeses.

Cheese Source	Presumptive Lactococci	Presumptive Thermophilic Cocci	Presumptive Lactobacilli	Coagulase-Negative Cocci	Enterococci	Yeasts	Molds
Producer 1	8.24 ± 0.16 ^ab^	7.16 ± 0.36 ^b^	8.40 ± 0.15 ^a^	5.34 ± 0.21 ^b^	6.81 ± 0.22 ^bc^	4.24 ± 0.75 ^a^	n.d.
Producer 2	8.66 ± 0.35 ^a^	7.95 ± 0.45 ^a^	8.50 ± 0.35 ^a^	5.91 ± 0.46 ^b^	7.57 ± 0.11 ^ab^	3.23 ± 0.32 ^a^	4.07 ± 0.58 ^a^
Producer 3	7.98 ± 0.20 ^b^	8.28 ± 0.17 ^a^	8.45 ± 0.09 ^a^	5.44 ± 0.10 ^b^	6.56 ± 0.12 ^c^	1.27 ± 1.47 ^b^	n.d.
Producer 4	8.41 ± 0.21 ^ab^	8.26 ± 0.31 ^a^	8.13 ± 0.20 ^a^	6.42 ± 0.22 ^a^	7.84 ± 0.73 ^a^	2.51 ± 0.70 ^ab^	n.d.

Values of viable counts are expressed as log cfu/g ± standard deviation of duplicate independent experiments. Within each column, overall means with different superscript letters are significantly different (*p* < 0.05). n.d., not detected.

**Table 5:** Identification of non-starter lactic acid bacteria (NSLAB) isolated from *Queijo Serra da Estrela* PDO cheeses.

Isolation Source	Isolate Code	Closest Relative	% Identity *	Accession Number **
Producer 1	SE1	*Enterococcus durans*	98.33%	NR_036922
	SE2	*Levilactobacillus brevis*	98.30%	NR_116238
	SE3	*Levilactobacillus brevis*	99.25%	NR_042438
	SE4	*Latilactobacillus graminis*	97.80%	NR_113922
	SE5	*Enterococcus durans*	98.65%	NR_036922
	SE6	*Enterococcus faecium*	99.30%	NR_114742
	SE7	*Enterococcus faecalis*	99.82%	NR_113902
Producer 2	SE8	*Lacticaseibacillus casei* group	98.66%	NR_025880
	SE9	*Lacticaseibacillus casei* group	97.89%	NR_025880
	SE10	*Lacticaseibacillus casei* group	99.27%	NR_117987
	SE11	*Levilactobacillus brevis*	98.99%	NR_116238
	SE12	*Enterococcus faecalis*	98.82%	NR_113902
	SE13	*Enterococcus faecalis*	98.96%	NR_113902
	SE14	*Enterococcus durans*	99.64%	NR_036922
	SE15	*Enterococcus lactis*	99.34%	NR_117562
	SE16	*Enterococcus durans*	99.35%	NR_036922
Producer 3	SE17	*Lacticaseibacillus casei* group	99.42%	NR_025880
	SE18	*Levilactobacillus brevis*	99.23%	NR_116238
	SE19	*Levilactobacillus brevis*	99.61%	NR_116238
	SE20	*Levilactobacillus brevis*	99.12%	NR_116238
	SE21	*Leuconostoc mesenteroides subsp. dextranicum*	99.23%	NR_113911
	SE22	*Enterococcus faecalis*	98.86%	NR_113902
Producer 4	SE23	*Latilactobacillus graminis*	98.81%	NR_042438
	SE24	*Levilactobacillus brevis*	99.40%	NR_116238
	SE25	*Lacticaseibacillus casei* group	97.80%	NR_025880
	SE26	*Latilactobacillus graminis*	99.28%	NR_042438
	SE27	*Leuconostoc mesenteroides subsp. dextranicum*	99.90%	NR_113911
	SE28	*Enterococcus durans*	97.45%	NR_036922
	SE29	*Enterococcus durans*	98.72%	NR_036922
	SE30	*Lacticaseibacillus casei* group	99.72%	NR_025880

* Percentage of identical nucleotides in the sequence obtained from the non-starter lactic acid bacteria (NSLAB) isolates and the sequence of the closest relative found in the GenBank database. ** Accession number of the sequence of the closest relative found by BLAST search.

**Table 6:** Semi-quantitative assessment of enzymatic activity of the pool of collected isolates from *Queijo Serra da Estrela* PDO cheeses.

Isolation Source	Isolate Code	Isolate	1	2	3	4	5	6	7	8	9	10	11	12	13	14	15	16	17
Producer 1	SE1	*Enterococcus durans*	0	0	2	1	0	0	0	0	0	0	2	1	0	0	0	0	0
	SE2	*Levilactobacillus brevis*	0	0	1	0	0	3	3	0	0	0	2	1	2	5	0	2	5
	SE3	*Levilactobacillus brevis*	0	0	2	1	0	4	4	1	0	0	5	3	3	5	3	2	5
	SE4	*Latilactobacillus graminis*	0	0	0	0	0	4	3	0	0	0	1	1	0	0	0	0	0
	SE5	*Enterococcus durans*	0	0	2	1	0	1	0	0	0	0	0	1	0	4	0	0	1
	SE6	*Enterococcus faecium*	0	0	2	1	0	1	0	0	0	0	1	1	0	5	0	0	1
	SE7	*Enterococcus faecalis*	0	1	2	1	0	0	0	0	0	0	3	1	0	0	0	3	1
Producer 2	SE8	*Lacticaseibacillus casei* group	0	0	3	3	0	4	5	1	0	1	2	4	0	3	0	2	1
	SE9	*Lacticaseibacillus casei* group	0	0	3	2	0	2	4	0	0	0	0	4	0	0	0	1	0
	SE10	*Lacticaseibacillus casei* group	0	0	2	1	0	4	5	1	0	0	3	4	0	1	0	2	1
	SE11	*Levilactobacillus brevis*	0	0	2	1	0	4	3	1	0	0	5	4	3	5	2	3	5
	SE12	*Enterococcus faecalis*	0	1	4	1	0	1	0	0	0	1	2	2	0	0	0	3	1
	SE13	*Enterococcus faecalis*	0	0	1	1	0	0	0	0	0	1	2	1	0	3	0	2	0
	SE14	*Enterococcus durans*	0	0	1	1	0	0	0	0	0	0	1	1	2	5	0	0	1
	SE15	*Enterococcus lactis*	0	0	2	2	0	2	0	0	0	0	1	1	0	1	0	0	2
	SE16	*Enterococcus durans*	0	0	2	2	0	1	0	0	0	0	1	1	2	5	0	0	2
Producer 3	SE17	*Lacticaseibacillus casei* group	0	0	3	3	0	5	5	1	0	1	3	4	0	1	0	4	3
	SE18	*Levilactobacillus brevis*	0	0	1	0	0	2	2	0	0	0	4	1	2	5	1	1	5
	SE19	*Levilactobacillus brevis*	0	1	2	1	0	5	3	1	0	0	5	5	1	5	3	2	5
	SE20	*Levilactobacillus brevis*	0	0	1	0	0	4	1	0	0	0	4	1	2	5	0	3	4
	SE21	*Leuconostoc mesenteroides*	0	0	3	1	0	2	0	1	0	0	1	1	0	3	0	0	1
	SE22	*Enterococcus faecalis*	0	0	2	1	0	1	0	0	0	1	1	3	0	0	0	2	1
Producer 4	SE23	*Latilactobacillus graminis*	0	3	0	0	0	5	4	j2	0	0	2	2	0	0	0	0	0
	SE24	*Levilactobacillus brevis*	0	0	2	1	0	5	4	3	0	0	5	5	5	5	4	4	5
	SE25	*Lacticaseibacillus casei* group	0	2	2	2	0	5	4	3	0	2	3	5	0	4	0	4	2
	SE26	*Latilactobacillus graminis*	0	1	0	0	0	4	4	0	0	0	1	1	0	0	0	0	0
	SE27	*Leuconostoc mesenteroides*	0	0	1	0	0	0	0	0	0	0	1	1	5	5	0	1	5
	SE28	*Enterococcus durans*	0	0	1	3	0	4	1	1	0	0	1	1	0	3	0	0	4
	SE29	*Enterococcus durans*	0	0	1	3	0	3	1	1	0	0	1	1	0	0	0	0	3
	SE30	*Lacticaseibacillus casei* group	0	0	1	3	0	5	3	1	0	0	2	2	0	3	0	1	2

1, control; 2, alkaline phosphatase; 3, esterase (C 4); 4, esterase lipase (C 8); 5, lipase (C 14); 6, leucine arylamidase; 7, valine arylamidase; 8, cystine arylamidase; 9, trypsin; 10, alpha-chymotrypsin; 11, acid phosphatase; 12, naphthol-AS-BI-phosphohydrolase; 13, alpha-galactosidase;14, beta-galactosidase; 15, beta-glucuronidase; 16, alpha-glucosidase; 17, beta-glucosidase. A value ranging from 0 to 5 was assigned, based on intensity of developed color: 0 corresponds to a negative reaction; 5 to a reaction of maximum intensity; 1, 2, 3, and 4 to intermediate reactions (3, 4 or 5 being considered as positive reactions).

**Table 7:** Volatile compounds (mean ± SD ^1^ of two cheese samples from the same batch) detected by SPME in the headspace of *Queijo Serra da Estrela* PDO cheeses.

Compound	CAS-NUMBER	Category	Flavour Note	Producer 1	Producer 2	Producer 3	Producer 4
ethanol	64-17-5	Alcohol	Alcohol, mild	4723 ± 1168	12892 ± 4021	7485 ± 1964	8199 ± 4395
propan-2-ol	67-63-0	Alcohol	rubbing alcohol	844 ± 47	4963 ± 5197	1350 ± 648	1005 ± 476
2-butanone	78-93-3	Ketone	Acetone, etheric	22638 ± 12768	11257 ± 721	15085 ± 4830	19462 ± 3181
butanal, 3-methyl	590–86-3	Aldehyde	Dark chocolate, malt, green	263 ± 227	62 ± 14	1257 ± 223	385 ± 514
isobutyl acetate	110-19-0	Ester	fruity, floral banana-like	76 ± 11	79 ± 78	53 ± 12	164 ± 112
3-hydroxybutan-2-one	5077-67-8	Ketone	Buttery	2067 ± 1026	2652 ± 1947	193 ± 253	541 ± 218
1-butanol, 3-methyl	123-51-3	Alcohol	Fruity, alcohol	21 ± 18	49 ± 65	31 ± 31	34 ± 13
propanoic acid, 2-methyl (isobutytic acid)	79–31-2	Acid	rancid butter	26 ± 6	32 ± 65	114 ± 87	60 ± 43
butanoic acid	107–92-6	Acid	Rancid, cheesy, putrid, sweaty	3135 ± 1923	2507 ± 2459	9613 ± 2312	10872 ± 4345
butanoic acid, 3-methyl (isovaleric acid)	503–74-2	Acid	Swiss, cheese, waxy, sweaty,	100 ± 57	176 ± 71	88 ± 66	97 ± 45
butanoic acid, 2-methyl	116–53-0	Acid	Fruity, sour, sweaty	109 ± 142	314 ± 5	1105 ± 644	719 ± 850
2-heptanone	110–43-0	Ketone	Floral, fruity	163 ± 55	60 ± 63	321 ± 104	153 ± 140
butanoic acid, 1-methylpropyl ester	819-97-6	Ester		38 ± 35	13 ± 11	563 ± 711	70 ± 60
benzaldehyde	100-52-7	Aldehyde	sweet, strong almond odour	184 ± 55	125 ± 106	127 ± 16	82 ± 42
ethyl hexanoate	123-66-0	Ester	Pineapple, apple powerful	11139 ± 8817	14680 ± 1815	60558 ± 24699	51008 ± 29977
hexanoic acid	142-62-1	Acid	Pungent, blue cheese, goat-like	227 ± 95	213 ± 157	246 ± 46	509 ± 262
phenylacetaldehyde	122-78-1	Aldehyde	pungent green floral and sweet odour of hyacinth type	182 ± 48	134 ± 62	215 ± 175	131 ± 129
heptanoic acid	111-14-8	Acid	rancid, sour, fatty odour	39 ± 11	50 ± 6	42 ± 5	55 ± 48
2-nonanone	821-55-6	Ketone	Musty, fruity, floral	188 ± 25	126 ± 15	184 ± 41	197 ± 63
octanoic acid	124-07-2	Acid	Goaty, waxy, soapy, rancid	9636 ± 7581	9715 ± 411	23449 ± 4690	18098 ± 8359
octanoic acid ethyl ester	106-32-1	Ester		1480 ± 987	3070 ± 1417	13440 ± 16802	6082 ± 5108
dodecane	112-40-3	Alkane		118 ± 59	169 ± 204	557 ± 581	251 ± 102
1,3-di-tert-butylbenzene	1014-60-4	Aromatic hydrocarbon		68 ± 59	367 ± 445	199 ± 139	261 ± 275
nonanoic acid	112-05-0	Acid	Coconut, fatty odour	63 ± 34	72 ± 30	304 ± 355	53 ± 48
decanoic acid	334-48-5	Acid	Rancid	3825 ± 2930	2184 ± 529	11532 ± 10468	17946 ± 17472
decanoic acid ethyl ester	110-38-3	Ester		1161 ± 664	1480 ± 496	5899 ± 7057	2118 ± 1010
dodecanal	112-54-9	Aldehyde	fatty odour	21 ± 4	19 ± 12	12 ± 4	40 ± 30
dodecanoic acid	143-07-7	Acid	like oil of bay	121 ± 98	45 ± 6	81 ± 45	191 ± 84
dodecanoic acid ethyl ester	106-33-2	Ester	fruity, floral	69 ± 33	149 ± 101	257 ± 288	136 ± 91

^1^ GC-FID peak areas [(pA × min) × 10^4^]. The most represented categories of compounds in the headspace of the *Queijo Serra da Estrela* PDO cheeses herein analysed were carboxylic acids (2-methyl propanoic, butanoic, 2-methyl butanoic, 3-methyl butanoic, hexanoic, heptanoic, octanoic, nonanoic, decanoic, dodecanoic) and esters (isobutyl acetate, 2-butyl butyrate, ethyl hexanoate, ethyl octanoate, ethyl decanoate, ethyl dodecanoate). Carbonyl compounds (2-butanone, 3-methyl butanal, 3-hydroxybutan-2-one, 2-heptanone, benzaldehyde, phenylacetaldehyde, 2-nonanone, dodecanal) and alcohols (ethanol, 2-propanol, 3-methyl butanol) were also detected. For each attribute, no significant differences among samples were observed.

## Data Availability

Data is contained within the article.

## Supplementary Materials

The following are available online at https://www.mdpi.com/article/10.3390/foods12010169/s1, Table S1: Incidence (%) of the bacterial taxonomic groups in *Queijo Serra da Estrela* PDO cheese samples according to producers. Table S2: Incidence (%) of the fungal taxonomic groups in *Queijo Serra da Estrela* PDO cheese samples according to producers.
