# Supplementary material for: Queijo Serra da Estrela PDO Cheese: Investigation into Its Morpho-Textural Traits, Microbiota, and Volatilome
Source: Foods. 2022 Dec 29;12(1):169. doi: 10.3390/foods12010169 (PMC9818377; doi:10.3390/foods12010169)
Supplement: Supplementary file 1 [file foods-12-00169-s001.zip › foods-2107730-supplementary.pdf]

Table S1. Incidence (%) of the bacterial taxonomic groups in *Queijo Serra da Estrela* PDO cheese samples according to producers.

ASVs	Producer 1	Producer 2	Producer 3	Producer 4
Acinetobacter guillouiae	0.05912072	0.02799858	0	0.35507628
Brevibacterium	0.81449714	0.03428571	0	0.05332385
Carnobacterium	1.10235223	0.05799503	1.60816572	0.31531948
Citrobacter	2.92450434	3.22588709	0.088767	0.28541773
Enterobacteriaceae	9.06370633	1.60220618	0.16718419	3.036131
Enterococcus	2.03783863	8.73728962	0.50473636	15.8841362
Gluconacetobacter	0.31019604	0.26741153	0.0413993	1.27833256
Lacticaseibacillus casei group	0.10084998	6.2180329	9.46629029	0.77385974
Lactiplantibacillus plantarum group	2.32547611	5.83007693	3.64423762	0.71128859
Loigolactobacillus coryniformis	0.03408316	2.20222038	3.89658619	4.1640759
Lactococcus garvieae	0.68768371	0.03942715	0	0.10520779
Lactococcus lactis	25.512839	30.6271133	25.6807807	2.85675322
Lactococcus piscium	5.19020265	2.29823174	36.5243568	42.3684758
Latilactobacillus sakei	21.8930091	10.2205705	1.93135645	10.6894878
Leuconostoc lactis	0.10919583	4.21559001	1.14162523	0.03554924
Leuconostoc mesenteroides	14.6505375	14.166486	4.91600955	10.7757522
Levilactobacillus brevis	1.72119973	0.76083797	1.41310606	0.16243532
Micrococcaceae	0.16137021	0.29684933	0.55488868	0.16387524
Serratia	4.14857545	6.37736063	7.99856226	4.267236
Staphylococcus equorum	0.18605789	0.24340869	0.20341347	0.44857301
Staphylococcus sciuri	0	0.51367026	0	0
Streptococcus	5.03281336	0.61796662	0.00676682	0.76563629

ASVs, amplicon sequence variants

Table S2. Incidence (%) of the fungal taxonomic groups in *Queijo Serra da Estrela* PDO cheese samples according to producers.

**ASVs**	**Producer 1**	**Producer 2**	**Producer 3**	**Producer 4**
Aspergillus	0.0000	0.0085	1.9418	0.0872
Candida boidinii	0.9618	15.4761	0.0000	0.1408
Candida sake	0.0017	0.2324	0.1378	0.4552
Cladosporium variabile	3.0565	0.7017	3.2920	2.8169
Clavispora lusitaniae	0.4127	0.2158	6.7394	1.0541
Cutaneotrichosporon curvatus	0.0000	0.0000	2.4974	1.4503
Debaryomyces hansenii	51.7352	15.1158	4.1062	11.0923
Galactomyces geotrichum	0.0000	30.3475	0.4306	0.0000
Geotrichum	0.9704	0.0000	0.0000	0.0000
Kurtzmaniella zeylanoides	9.1411	19.6062	0.0000	0.0000
Lachancea thermotolerans	0.3177	0.0000	1.9979	1.0039
Metschnikowia fructicola	0.0000	0.0000	8.1079	2.2409
Naganishia albidosimilis	0.0000	9.9964	0.0000	0.0000
Nakazawaea wickerhamii	0.1815	0.2111	0.3445	0.3215
Penicillium	0.5776	2.4622	0.1550	0.0000
Pichia fermentans	0.2335	0.1436	0.7234	4.3336
Protomyces inouyei	0.0035	0.7666	5.0988	0.0000
Starmerella	0.0000	0.5422	17.6603	34.0855
Symbiotaphrina	0.0350	0.0408	2.2378	0.7687
Ustilago	0.5346	0.8299	0.3961	1.2028
Vishniacozyma victoriae	23.9039	1.0897	0.1895	2.0140

ASVs, amplicon sequence variants
